# Proteomic and Targeted Lipidomic Analyses of Fluid and Rigid Rubber Particle Membrane Domains in Guayule

**DOI:** 10.3390/plants13212970

**Published:** 2024-10-24

**Authors:** Joshua J. Blakeslee, Eun-Hyang Han, Yun Lin, Jinshan Lin, Seema Nath, Liwen Zhang, Zhenyu Li, Katrina Cornish

**Affiliations:** 1 Department of Horticulture and Crop Science, Ohio Agricultural Research and Development Center (OARDC), The Ohio State University, Wooster, OH 44691, USA; han.637@buckeyemail.osu.edu (E.-H.H.); lin.1418@buckeyemail.osu.edu (Y.L.);; 2 Laboratory for the Analysis of Metabolites from Plants (LAMP), The Ohio State University, Columbus, OH 43210, USAnaths@uthscsa.edu (S.N.); 3 Campus Chemical Instrumentation Center (CCIC), The Ohio State University, Columbus, OH 43210, USA; zhang.287@osu.edu; 4 EnergyEne, Inc., 5659 Canaan Center Road, Wooster, OH 44691, USA; 5 Department of Food, Agricultural and biological Engineering, Ohio Agricultural Research and Development Center (OARDC), The Ohio State University, Wooster, OH 44691, USA; 6 U.S. Arid Land Agricultural Research Center, Maricopa, AZ 85138, USA

**Keywords:** guayule, membrane, lipidomics, proteomics, rubber particle membranes, rubber transferase complex

## Abstract

Rubber (*cis*-1,4-polyisoprene) is produced in cytosolic unilamellar vesicles called rubber particles (RPs), and the protein complex responsible for this synthesis, the rubber transferase (RTase), is embedded in, or tethered to, the membranes of these RPs. Solubilized enzyme activity is very difficult to achieve because the polymerization of highly hydrophilic substrates into hydrophobic polymers requires a polar/non-polar interface and a hydrophobic compartment. Using guayule (*Parthenium argentatum*) as a model rubber-producing species, we optimized methods to isolate RP unilamellear membranes and then a subset of membrane microdomains (detergent-resistant membranes) likely to contain protein complexes such as RTase. The phospholipid and sterol composition of these membranes and microdomains were analyzed using thin-layer chromatography (TLC) and liquid chromatography tandem mass spectroscopy (LC-MS/MS). Our data indicate that RP membranes consist predominantly of phosphatidic acid-containing membrane microdomains (DRMs or “lipid rafts”). Proteomic analyses of guayule RP membranes and membrane microdomains identified 80 putative membrane proteins covering 30 functional categories. From this population, we have tentatively identified several proteins in multiple functional domains associated with membrane microdomains which may be critical to RTase function. Definition of the mechanisms underlying rubber synthesis will provide targets for both metabolic engineering and breeding strategies designed to increase natural rubber production in latex-producing species.

## 1. Introduction

Natural rubber (NR; *cis*-1,4-polyisoprene) is a vital strategic resource with innumerable industrial uses (more than 50,000 products) [1]. NR is a secondary metabolite and terminal carbon sink that is generated via the enzymatic polymerization of isopentenyl pyrophosphate in at least 2500 plant species [2]. Currently, commercial natural rubber production occurs solely in *Hevea brasiliensis* Műll, Arg. (para or Brazilian rubber tree) plantations, over 90% of which are located in Southeast Asia [3,4]. However, due to the genetic uniformity of the *H. brasiliensis* stands, natural rubber plantations are increasingly threatened by diseases, including the fatal South American Leaf Blight (SALB) [3] and the serious *Pestalotiopsis* and *Neofusicoccus* blights [5], which infected >500,000 ha of rubber trees in Southeast Asia in 2019. In addition to these disease problems, *H. brasiliensis* trees are being replaced by high-value, lower-maintenance crops, such as oil palm trees [4,6,7], and a current global deforestation moratorium limits replacement planting on newly cleared forest land. These difficulties in production, combined with increased global rubber consumption (particularly in growing economies in South America, East Asia, and Southeast Asia) and extreme weather events, led to a 10% decline in rubber production in 2020 (1.4 million metric tons) (https://www.bloomberg.com/news/articles/2021-04-13/rubber-scarcity-creates-new-headache-for-beleaguered-automakers?srnd=premium&sref=KFo8mQVH; accessed on 15 October 2024). Increased demand for personal and medical protective equipment (especially gloves and masks) required an additional one million tons of latex at peak usage at the height of the COVID-19 pandemic.

Rubber particles (RPs) are specialized rubber-producing organelles found in the cytosol of either specialized laticifer cells, like those found in *H. brasiliensis, Ficus elastica,* Roxb. Ex Hornem (rubber fig or Indian rubber tree), and *Taraxacum kok-saghyz*, Rodin (rubber or Russian dandelion), or parenchyma cells, as in both *Parthenium argentatum* Gray (guayule) and *Ericameria nauseosa* (http://www.theplantlist.org/tpl1.1/search?q=Ericameria+nauseosa; accessed 15 October 2024) Pall. Ex Pursh, formerly *Chrysothamnus nauseosus* (common or grey rabbitbrush) [8,9,10,11,12,13]. RPs are microscopic, cytosolic, usually spherical or oblately spheroid, rudimentary organelles defined by unilamellar membranes. The physiological role of RPs in plants has not yet been clearly identified, although rubber production is often associated with environmental or abiotic stress [14]. Rubber production in *T. kok-saghyz* and *P. argentatum* is induced and enhanced by the cold in temperate regions and may limit low-temperature damage to cellular organelles [15,16,17]. In tropical species, including *H. brasiliensis* and *F. elastica,* rubber production is often stimulated by wounding stress and may help to seal wounds and reduce pathogen infection and insect predation [3,14,18,19].

The outer hydrophilic surface of the RP unilamellar membrane is negatively charged, which inhibits rubber particle agglomeration in the cytosol, as the aggregate charge state causes repulsion between individual rubber particles. When mature, RPs are often packed into the vacuoles, a normally acidic compartment where they may or may not coagulate [20]. The negative charge of the rubber particle membrane is likely the result of its unique sterol and phospholipid composition (in particular, a high concentration of phosphatidic acid [14]) and by carboxyl anions [11]. The RP membrane also contains rubber transferase (RTase) complexes composed of specialized proteins responsible for rubber biosynthesis [12,14]. In the rubber-producing tissues and cells of all species studied so far, rubber polymerization is catalyzed by RTase proteins localized at the RP surface, some with integral membrane moieties, and the hydrophobic rubber chains are compartmentalized into the interior of the RP as they are elongated to sizes often > 1 Mg.mol^−1^ [3,10,11,21]. The unilamellar biomembrane surrounding the RP rubber core [10,11] is composed of a species-specific complement of proteins and lipids [12]. Both rubber particle size and the molecular weight of the rubber polymers produced are species-specific and can be altered by environmental conditions [11,19,22,23].

Several individual rubber particle proteins appear to play roles in rubber biosynthesis, chain length regulation, polymerization, and substrate binding [24]. However, the actual RTase complex composition and structure remains unclear, and all but one study [25] have failed to produce rubber polymer in the absence of RPs, the sites of rubber synthesis in vivo, indicating that, to date, the identity of the complete complement of proteins necessary to assemble a complete RTase complex and synthesize rubber remains unknown [24].

In this study, rubber particle membranes and a subset of rigid membrane microdomains (detergent-resistant membranes, “DRMs”, also referred to as “lipid rafts”) were isolated from intact, enzymatically active RPs of *P. argentatum*. DRMs are areas of increased structural organization in membranes and exhibit distinctive lipid profiles, including increased sterol composition and levels of phosphatidic acid. The composition of DRMs results in decreased membrane fluidity in these membrane microdomains, a structural characteristic which stabilizes and facilitates the formation of multi-protein complexes. Because of this, we hypothesized that, if present in RPs, the DRM fraction of RP membranes would be a preferential site of localization for the RTase complex. To investigate this hypothesis, the lipid and protein profiles of RP membranes and DRMs were characterized and interpreted.

## 2. Results

### 2.1. Isolation of Membrane and DRM Fractions from P. argentatum Washed Rubber Particles (WRPs)

**Figure 1:**
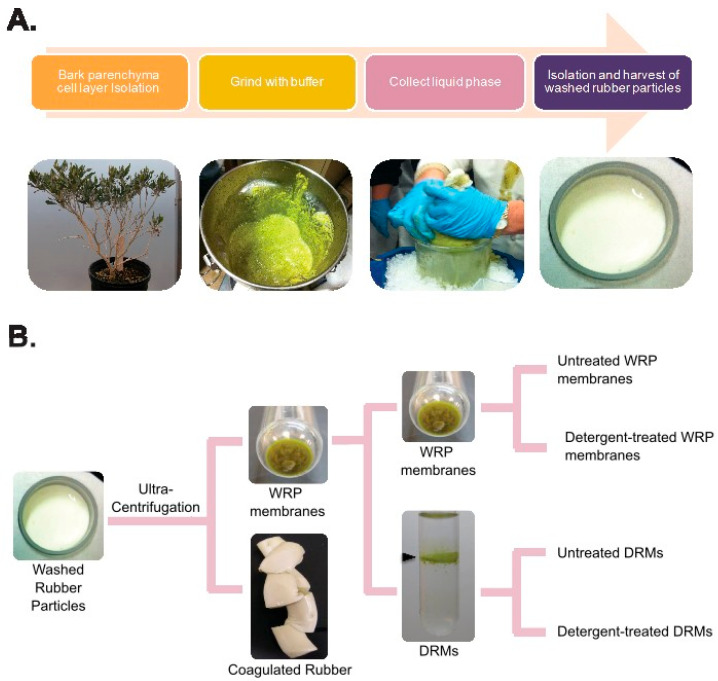
Isolation of rubber particle membrane and membrane microdomains. (**A**). Isolation of enzymatically active washed rubber particles from guayule bark parenchyma tissues. Guayule bark was collected from stems and defoliated branches of harvested shrub. Tissues were ground and WRPs were isolated via liquid extraction and washed using low-speed centrifugation (<10,000× *g*). (**B**). Isolation of membranes and detergent-resistant membrane microdomains from guayule WRPs. Membranes and DRMs were prepared as detailed [27]. Following isolation, WRPs were resuspended in membrane extraction buffer, and then membranes were isolated by subjecting WRPs to high-speed ultracentrifugation at 100,000× *g*, 4 °C for 50 min. During this step, rubber particle membranes precipitated to the bottom of the tube, while the latex fraction floated to the top. The latex fraction was coagulated with glacial acetic acid and removed from each sample. The membrane fraction was weighed, then resuspended and split into two sub-fractions. One sub-fraction (membrane) was washed, re-precipitated, weighed, and sent for proteomic and lipid profiling analyses. The other sub-fraction (detergent resistant membrane microdomains; DRMs) was weighed, resuspended, treated with 0.1% (*v*/*v*) Triton-X 100, layered onto a sucrose gradient, and subjected to a second round of ultracentrifugation. DRMs, which floated to the interface between the sucrose layers (black arrow on the image of the “DRM” tube), were collected, washed, precipitated, weighed, and sent for proteomic and lipid profiling analyses.

To collect enough enzymatically active WRPs to generate sufficient membranes for downstream analyses, WRPs were prepared in bulk from large (kilogram-level) lots of guayule tissue (Figure 1A). Four membrane preparations (i.e., independent biological replicates) were successfully isolated from WRPs in the winter of 2013–2014, and an additional five membrane preparations were isolated from WRPs in the winter of 2014–2015 (Figure 1B). Total membranes (i.e., the membrane fraction isolated from WRPs) accounted for between 0.51 and 2.53% of the total mass of RPs isolated in the winter of 2013/2014 and between 1.31 and 1.60% in the winter of 2014/2015. Isolated membranes were treated with the detergent Triton-X100, and detergent-resistant membrane microdomains (DRMs, also referred to as “lipid rafts”) were purified via sucrose density gradient flotation, a method previously utilized to isolate membrane DRMs from *Arabidopsis thaliana* microsomal membrane preparations [26]. WRP membrane microdomains resistant to Triton-X100 detergent solubilization behaved similarly to DRMs isolated from *A. thaliana*. WRP DRMs localized to the interface between the layers of 0.15 M and 1.4 M sucrose following density gradient centrifugation (Figure 1B). RP membranes made up between 1.310% and 2.533% of the total RP mass in the Year 1 RP sample set and between 1.604% and4.315% of the total RP mass in the Year 2 RP sample set. DRMs made up a substantial part of the total membrane composition of the RP membranes. DRMs accounted for between 54.41% and 84.83% of the RP membrane mass in Year 1 samples and between 39.45% and 90.60% of the rubber particle membrane mass in Year 2 (Table 1).

### 2.2. Phospholipid and Sterol Analyses

**Figure 2:**
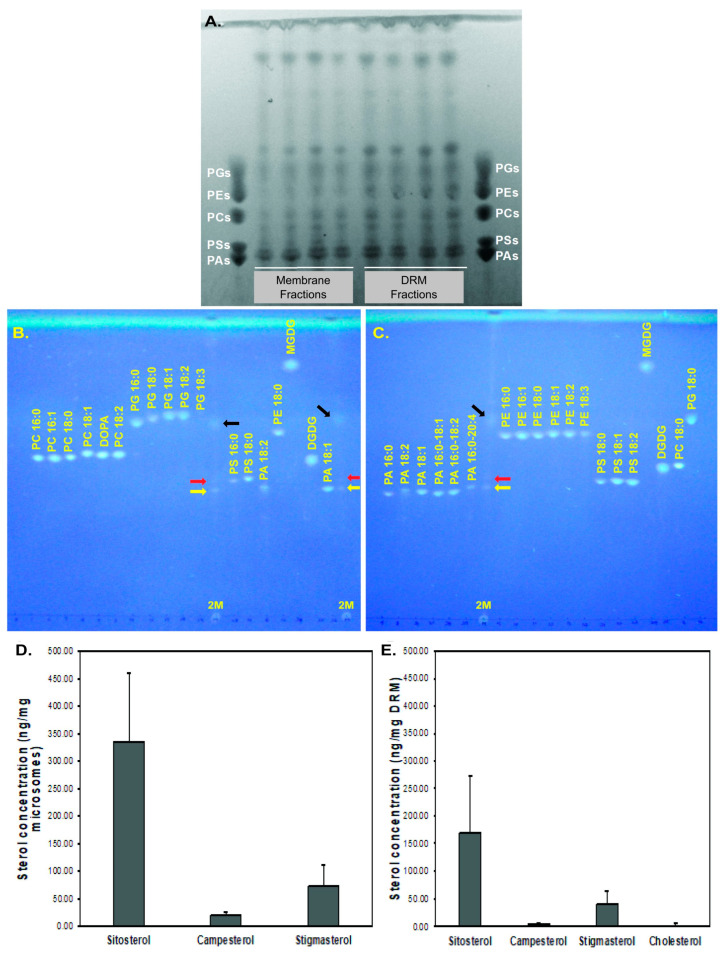
Thin-layer chromatographic analyses of membrane and detergent-resistant membrane microdomain fractions isolated from rubber particles. (**A**)**.** TLC of RP membrane and DRM fractions. Samples were run against a mixture of phospholipid standards and phosphatidic acids. Lipids were imaged using primulin. (**B**,**C**). TLC of a representative RP membrane fraction (1 µg standards loaded and 6 µL guayule sample per TLC plate). Glycerophospholipids were resolved first by head-group and then, to a lesser degree, by chain length and saturation. TLC analyses indicated the most prevalent components of the RP and DRM samples were phosphatidic acids (PA 18:1; yellow arrow), phosphatidylglycerol (PG 18:0; black arrow), and potentially phosphatidylserine (PS 18:0; red arrow). The assay was repeated 4 times and representative data are shown here. (**D**,**E**). LC-MS/MS quantification of sterols in guayule membrane (**D**) and detergent-resistant membrane microdomain (**E**) fractions. Sterols were extracted using chloroform:methanol (70:30) and quantified using QQQ LC-MS/MS. Retention times and mass transitions for sterols were determined using authentic standards. Quantifications were performed using deuterated authentic standards.

TLC-based profiling of rubber particle membrane and DRM fractions identified phosphatidic acid (PA), phosphatidylcholine (PC), phosphatidylethanolamine (PE), phosphatidylglycerol (PG), and phosphatidylserine (PS) and revealed several unidentified/unknown bands (Figure 2A; Supplementary Table S1). The intensity of PA bands was notably higher than that of other phospholipids in individual samples (Figure 2A). TLC and LC-MS/MS analyses detected dioleoyl phosphatidic acid (DOPA, PA 18:1), PA16:0-18:2. PC18:1, PC18:2 (DLPC; 1,2-dilinoleoyl-sn-glycero-3-phosphocholine), PC16:0 (DPPC, 1,2-dipalmitoyl-sn-glycero-3-phosphocholine), PE16:0, PE16:1, and DOPE (1,2-dioleoyl-sn-glycero-3-phosphoethanolamine) in RP membrane fractions (Figure 2A,B). Similarly, DOPA (PA 18:1), PA16:0-18:2. PC18:1, PC18:2 (DLPC), PE16:0, and PE16:1 were detected in DRM fractions (Figure 2). Thus, PA 18:1, PA16:0-18:2. PC18:1, PC18:2, PE16:0, and PE16:1 were present in both RP membrane and DRM fractions (Figure 2A; Supplementary Table S1). PC16:0 was only detected in RP membrane fractions. PA 18:1, PA16:0-18:2. PC18:1, and PC18:2 were detected in all biological replicates of RP membrane and DRM fractions, while PC16:0, PE16:0, and PE16:1 were detected in some, but not all replicates.

Similarly, LC-MS/MS sterol analyses identified sitosterol as the predominant sterol in RP membrane fractions (average concentration of 335.21 ± 124.68 ng/mg microsomal membrane), followed by stigmasterol (72.60 ± 39.78 ng/mg) and campesterol (19.60 ± 5.54 ng/mg) (Figure 2D,E). Sitosterol (present at 169.49 ± 102.55 ng/mg DRM) was also the predominant sterol in the DRMs, followed by stigmasterol (40.73 ± 24.11 ng/mg), campesterol (4.15 ± 1.85 ng/mg), and cholesterol (which was not detected in the RP membrane fraction) at a concentration of 2.25 ± 3.89 ng/mg (Figure 2D,E). Overall, the sterol composition of DRMs was very similar to that of the RP membrane fractions, consistent with the hypothesis that DRMs make up a large percentage of the total WRP membrane. Sterols are considered essential for the formation and maintenance of DRMs [26]. Interestingly, several proteins involved in specialized lipid metabolism, including a putative mono- and diacyl-glycerol lipase and a fatty acid lipoxygenase 2 enzyme, were identified in our proteomic analyses, as detailed below.

### 2.3. Proteomic Analyses

**Figure 3:**
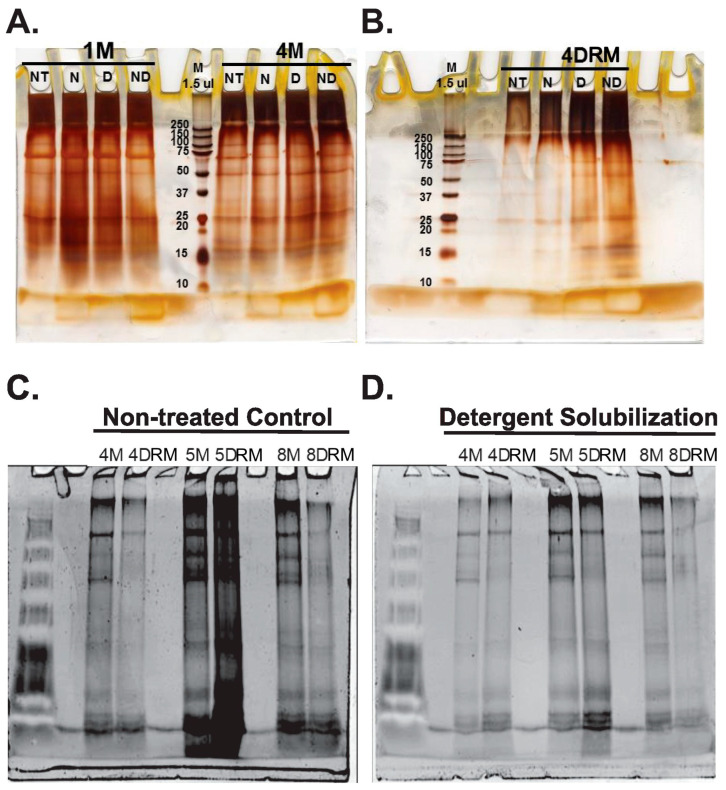
Isolation of proteins from rubber particle membranes. (**A**,**B**). Detergent solubilization of proteins from guayule RP membrane (**A**) and detergent-resistant membrane microdomain (**B**) fractions. Proteins were separated using SDS-PAGE and visualized via silver staining. NT: non-treated control; N: NP-40 substitute (2% *v*/*v*); D: deoxycholate (0.5% *v*/*v*); ND: NP-40 substitute and deoxycholate (0.25% each, *v*/*v*). (**C**,**D**)**.** Separation of WRP peptides for sequencing. Three paired biological replicates of both RP membrane (4M, 5M, 8M) and detergent-resistant membrane microdomain (4DRM, 5DRM, 8DRM) fractions were prepared, separated via SDS-PAGE, and visualized using SYPRO staining. (**C**). Non-treated samples. (**D**). Samples treated with 0.5% deoxycholate.

Protein levels in the membrane preparations from the 2013/2014 winter harvest ranged from 41.61 to 78.50 µg/µL in RP membrane fractions and from 19.05 to 35.82 µg/µL in DRM fractions (Table 2). Proteins in RP membrane and DRM fractions were separated using SDS-PAGE and visualized by silver staining. At least 10 distinct protein bands from membrane fractions and at least 6 bands from DRM were clearly visualized. Next, a series of detergents was tested to determine their ability to solubilize proteins from RP membrane and DRM fractions to aid in proteomic sequencing. Deoxycholate was best able to solubilize proteins from both membrane and DRM fractions (Figure 3C) and was used to extract proteins from RP membrane and DRMs for proteomic sequencing (Figure 3A).

Both detergent-solubilized and non-solubilized RP membrane and DRM samples were separated via SDS-PAGE (Figure 3B) and trypsin-digested, and the resulting tryptic peptide fragments were sequenced via LC-MS/MS. The sequences obtained were BLASTed against both the NCBI database (National Center for Biotechnology Information; https://blast.ncbi.nlm.nih.gov/Blast.cgi; accessed on 1 May 2024) and a *P. argentatum* transcriptome library generated by EnergyEne, Inc., Wooster, OH (BLAST results shown in Supplementary Table S2). A total of 403 fragments were sequenced from RP membrane and DRM fractions. From this data set, 170 fragments matched sequences found in the *P. argentatum* transcriptome library, and 71 fragments were found only in the NCBI database. Overall, and including repeated matches for the same peptide fragment, BLAST comparisons resulted in 1540 matches against the *P. argentatum* transcriptome library and 1327 matches against the NCBI database. The BLAST data showed almost identical results from both RP membrane and DRM fractions, again supporting the hypothesis that there is considerable overlap in these protein populations and that DRMs make up a significant portion of the total RP membrane. Ultimately, our analyses allowed for the putative identification of approximately 80 proteins, which we clustered into functional groups and discuss below.

#### 2.3.1. Peptides Putatively Involved in ATP Homeostasis

Interestingly, proteins involved with ATP metabolism and homeostasis were, in terms of the number of putative peptides identified, the largest group of proteins detected in RP membranes and membrane sub-domains. Specifically, our analyses identified seven peptides involved with ATP synthesis (including several members of ATP-synthase complexes), seven peptides identified as either vacuolar ATPases or components of vacuolar ATPase complexes, and two peptides functioning as either plasma membrane ATPases or components of plasma membrane ATPases. In addition to proteins involved in ATP synthesis or metabolism, our analyses also identified an ATP/ADP carrier protein and an adenylate transporter, both of which have the potential to impact ATP metabolism.

#### 2.3.2. Putative Transport Proteins

Following ATP metabolism, the next largest cluster of putative peptides was those potentially involved in the transport of a range of ions and solutes across the RP membrane. Our BLAST analyses identified a voltage-dependent anion channel; an aquaporin; a peptide with homology to the TOC75 chloroplast membrane translocon complex protein; and two porin-type proteins, with homologies to the outer membrane porin OprF and mitochondrial outer membrane porins. An ortholog of NtPDR1 was also identified in RP membrane fractions. NtPDR1 transports several diterpenes across the plasma membrane in tobacco [27]. Interestingly, proteomic data also indicated the presence of a pyrophosphate-activate proton transporter capable of hydrolyzing pyrophosphate residues to power the transmembrane movement of protons. Finally, it should be noted that several proteins identified in Section 2.3.1 as being involved in ATP homeostasis also function as transporters, specifically the vacuolar proton ATPase and plasma membrane proton ATPase proteins, both of which use the energy generated by the hydrolysis of ATP to transport protons across a membrane. In addition to these ATPase proteins, we also identified an ATP/ADP anti-porter, which could also contribute to ATP/ADP homeostasis.

#### 2.3.3. Putative Redox-Associated Proteins

An additional group of peptides isolated from RP membranes and membrane microdomains were peptides associated with redox reactions. In this cluster were three peptides with homology to cytochrome f, c1, and b complex members. Additionally, this group contained a NADH-ubiquinone oxidoreductase protein and an NADH dehydrogenase subunit.

#### 2.3.4. Cytoskeletal Proteins and Peptides Putatively Associated with Protein Folding

Given the close association of membranes enclosing and defining organelles with the cytoskeleton, we anticipated identifying several cytoskeletal proteins in our proteomic screen of RP membranes and membrane microdomains. Our analyses identified several such proteins, including actin, beta-tubulin, and the myosin heavy chain. A protein with homology to the clathrin heavy chain was also identified, as was a potential NADH dehydrogenase subunit. In addition to cytoskeletal proteins, our analyses also identified several peptides with roles in protein folding, including a heat-shock cognate 70 kDa protein and a peptide with homology to 18.5 kDa class I HSPs.

#### 2.3.5. Proteins Associated with Rubber Synthesis and Putative Members of the Rubber Transferase Complex

The most abundant protein found in RP membrane and membrane microdomain fractions was the rubber particle protein (RPP) [28], previously identified as sharing homology with P450 allene oxide synthases [29]. However, it is unlikely that this peptide is serving as an allene oxide synthase in the RP membrane, and it is more probable that this protein is exerting an alternative function in rubber synthesis. Additionally, our proteomic screening identified a long-chain acyl-CoA synthetase which may also play a role in either providing substrate for long-chain fatty acid synthesis in rubber particles or the transport of long-chain fatty acid acyl-CoA conjugates. Acyl-CoA synthetases activate fatty acids by linking them to Co-enzyme A moieties, allowing the fatty acid chains to be modified or transported. In *A. thaliana*, long-chain acyl-CoA synthase enzymes play roles in the synthesis of cuticular wax (CER8) and the fatty acids and triacylglycerides present in the oil bodies of seeds, as well as a range of stress responses [30,31]. Interestingly, we also identified an inorganic pyrophosphatase which may be involved in metabolically balancing rubber synthesis at the RP membrane (see below, Section 3). Finally, it should be noted that, given their abundance in RP membranes, it is possible, and indeed likely, that many of the other putative proteins identified in the groups above may also function either directly or indirectly in rubber synthesis as members of the membrane-bound RTase. Examination of the relative abundance of the peptides allowed us to identify six types of protein not previously identified in RP membranes as being most abundant (i.e., most well-represented in the population of peptides sequenced) in both rubber particle membranes and membrane microdomains. These proteins include: one or more allene oxide synthase-like proteins (most likely representing the rubber particle protein RPP), a vacuolar ATPase/plasma membrane proton ATPase, a pyrophosphate-energized proton pump, a voltage-dependent ion channel, and an ion exchange transporter (most likely a sodium or potassium/calcium exchanger).

## 3. Discussion

In this study, detergent-resistant membrane microdomains were successfully isolated from enzymatically active WRP membranes. The DRM fractions accounted for a very high portion of the total RP membrane fraction (Table 1). This finding is supported not only by our percent mass calculations, but also by the similar levels of sterols found in DRM and RP total membrane fractions (Figure 2D).

Previous studies analyzed the lipid and sterol composition of RPs primarily via solvent-based extractions of RPs followed by TLC and GC-MS analysis [12]. While these results provided insight into the lipid composition of RPs from different species, the solvent-based extractions used will almost certainly disrupt membrane two- and three-dimensional architecture, and/or under-represent sterols present in the extract. Further, this earlier study performed TLC and FAME (fatty acid methyl ester) analyses separately, making it impossible to state conclusively which fatty acid chains are associated with a given phospholipid head group (i.e., which species of phospholipids are present in the rubber particle membrane). Additionally, this type of GC-MS analysis cannot differentiate fatty acids released from membrane phospholipids (a relatively small population) from free fatty acids potentially extracted from the core of the rubber particle by the organic solvent, further complicating interpretation of the results. In contrast to these approaches, the TLC and LC-MS/MS methods used in this study focused on intact membranes and membrane microdomains isolated from enzymatically active rubber particles, making it better suited to accurately identify the phospholipids and sterols present in RP membrane and DRM fractions. Finally, it should be noted that the current analyses did not include quantification of free fatty acids, the bulk of which should remain in the center of the rubber particle and thus be subtracted from our analyses as we separated membrane fractions from latex.

Sitosterol, stigmasterol, and campesterol were detected in both RP membrane and DRM fractions. Interestingly, cholesterol, which is found less frequently in plant membrane samples than in microbial or animal membranes, was only detected in DRM fractions, and in these fractions at levels just above the limit of detection. The detection of cholesterol in DRM fractions and not in RP membrane fractions indicates that its concentration is greater in the DRM fractions than the rest of the RP membrane, but does not prove that it is completely absent in non-DRM areas. As noted above, DRMs contain higher levels of sterols than do normal membrane fractions, and these sterols are credited with contributing significantly to the ordered structure and “stiffness” of DRMs [32].

The need to understand the lipid composition of RP membranes is becoming increasingly clear and may prove key to the reconstitution of RTase activity in liposomes. Recent studies have attempted to produce high-molecular-weight natural rubber through reconstitution of rubber synthesis proteins in heterologous expression systems [33,34,35]. These reconstituted rubber biosynthetic apparatuses included *cis*-prenyl transferases (CTPs), rubber elongation factor (REF), and/or CTP-REF binding proteins, and were expressed in cell-free expression systems, yeast, yeast two-hybrid systems, cleaned native RP membranes, and nanodisc reconstitution systems [33,34,35]. However, each of these attempts failed to synthesize high molecular weight rubber chains, and even HRP1 did not show activity when a HRP1, REF, and CTP-REF complex was reconstituted using a cell-free expression system [33,34]. HRP1 did show activity, however, when added back to RPs previously inactivated by detergent [33]. These data support the hypothesis that maintenance of the proper lipid and sterol environment is essential to proper functioning of the RTase in addition to the polar/non-polar interface. Our data provide more definitive support for the role of membrane architecture in rubber particle physiology and describe, for the first time, the extensive presence of detergent-resistant membrane microdomains/lipid rafts in the unilamellar rubber particle membrane. Both the pervasiveness of DRMs in the RPM membrane and the high concentrations of phosphatidic acid (particularly the 18:0 species of PA) and sterols in these membrane DRMs make it likely that RTase is located in PA-rich rigid microdomains. Also, it is known that environmental stimuli, such as cold, induce the phospholipase D family (PLD) and result in increased PA production [36]. High PA levels in RPs isolated from cold-induced guayule shrub indicates that PA is linked to the generation of new RPs and leads ultimately to increased rubber synthesis.

Proteomic analyses of RP membrane and DRM fractions led to the putative identification of several potential members of RTase. The two small proteins known to be directly involved in substrate binding [37] were not found in the proteomic characterization, but this is not surprising because these RTase-associated proteins are not covalently bound and can be leached off enzymatically active RPs, so these proteins were likely “lost” to the soluble fraction when isolating rubber particle membrane fractions. The most abundant protein in our fractions was the 52 kDa RPP, identified as a P450 allene oxide synthase homolog [29,38]. However, the presence of the additional peptides indicates that other proteins not directly involved in rubber chain polymerization may serve as critical components of the membrane-bound RTase complex. Functional analyses of these peptides allowed for the identification of several novel putative members of the RTase complex, including: one or more allene oxide synthase-like proteins (i.e., most likely RPP-like proteins and orthologs), a vacuolar ATPase, a plasma membrane proton ATPase, an inorganic pyrophosphatase, a voltage-dependent ion channel, and a putative ion exchange transporter (likely sodium or potassium/calcium). These proteins are in addition to the two very small substrate binding proteins and one large scaffold protein previously identified [24,37] and, as part of an RTase complex along with the proteins catalyzing rubber chain elongation, may allow stabilization of rubber synthesis and the generation of long-chain isoprenoids. Interestingly, aside from proteins potentially involved in terpenoid synthesis and fatty acid synthesis (proteomic analysis indicated the presence of an acetyl-CoA synthase enzyme) or modification/activations reactions, the primary proteins present in membrane fractions (discounting cytoskeletal proteins such as actin) are involved with ATP homeostasis, ion transport, hydrogen extrusion, and oxidation/reduction reactions. In other words, these proteins have the potential to extrude metabolic by-products generated by terpenoid synthesis from the interior of the rubber particle. While we were somewhat surprised by the finding of ATPase components and transport proteins in RP membranes, the extensive washing of RPs prior to RP membrane separation should have removed contaminating fragments of other cellular membranes, as well as any non-integral membrane peptides associated with the RPs. The finding of larger transport-related proteins in RP membranes is not entirely unreasonable. These proteins may be targeted to the RP membrane during ontogeny (i.e., as the nascent RP is being formed from the endoplasmic reticulum), while the membrane is still bilamellar in nature [8]. Alternatively, since mature RPs are stored in the vacuole [8,10,20], it is possible that some tonoplast-associated peptides are transferred to the RP membrane as the RPs move into the vacuole (a phagocytotic process involving extensive RP membrane-tonoplast interactions) [8,20].

**Figure 4:**
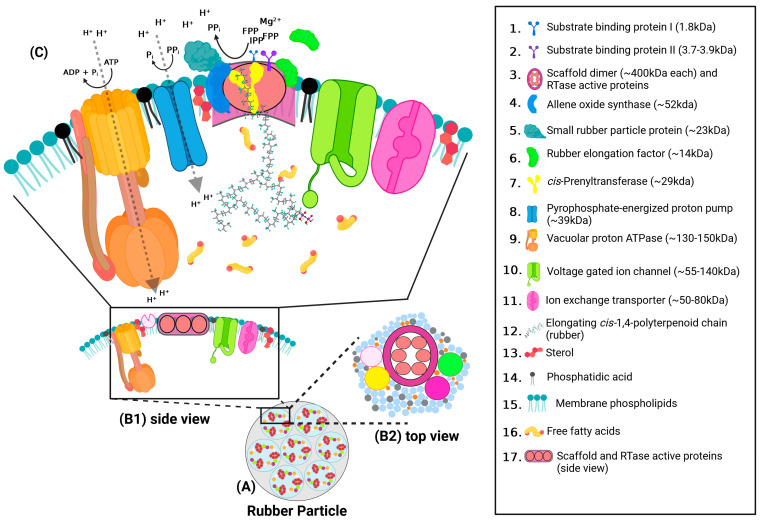
Model of a putative rubber transferase (RTase) complex embedded in detergent-resistant membrane microdomains located extensively throughout the unilamellar RP membrane. Identities of individual RTase members are detailed in the inset to the right of the model. (**A**). Top-down view of RTase complexes in the RP membrane. RTase complexes are located in detergent-resistant membrane microdomains (DRMs; light-blue circles), which make up a significant portion of the RP membrane. Non-DRM membrane is represented as a gray background. (**B1**,**B2**). Magnified views of a single RTase complex embedded in a DRM. (**B1**). A side view showing the RTase complex position in the RP DRM microdomain. Sterols and phospholipids crucial to the formation and maintenance of this membrane microdomain are highlighted as red sterol backbones and dark gray phospholipids, respectively. (**B2**). Top-down view of the putative RTase complex in the DRM. Sterols and phosphatidic acid are highlighted as orange and gray circles, respectively. The site of active rubber synthesis formed by catalytic sites on the RTase scaffold serves as a “functional pore” in the membrane in which substrate is converted into rubber chains, which are fed into the interior of the particle. (**C**). Detailed view of the function of the putative RTase complex in a DRM microdomain. The large dimeric scaffold holds the proteins critical to making the rubber polymer in place and forms the hydrophobic channel to the particle’s interior. As the synthesis of rubber molecules proceeds at the catalytic site of the RTase complex, the hydrogen and pyrophosphate ions generated during catalysis gradually accumulate in the inner surface of the membrane adjacent to the allene oxide synthase-like/RPP proteins, potentially acidifying the environment and/or inhibiting or halting polymerization. To prevent this from occurring, RTase-associated pyrophosphate-energized proton pumps hydrolyze excess pyrophosphate and remove excess protons into the interior of the RP. ATP-ases and ADP/ATP antiporters also function in maintaining proton and ATP homeostasis in the RP. Other ion channels function to maintain homeostasis of calcium, magnesium, or other cations in the area around the RTase complex.

Based on the proteins identified in our proteomic screening, we propose a more complete model of rubber biosynthesis (Figure 4) in which RTase complexes are embedded in lipid rafts/DRMs distributed across (and making up a large percentage of) the unilamellar membranes of rubber particles (Figure 4A). In this model, the core RTase enzyme and protein components are similar across species, but the fatty acid and sterol composition of the DRMs, in which the RTase complex and associated proteins are localized, are species-specific [12]. Our proteomic analyses indicated the presence of several enzymes involved in lipid and fatty acid metabolism associated with RP membranes and DRMs. It is likely that these enzymes function in rubber particle growth rather than rubber biosynthesis. As the rubber particle expands, hydrophobic membrane components and proteins incorporate into the particle. Enzymes involved in fatty acid metabolism and transport of long-chain fatty acids may be involved in maintaining membrane and DRM structure. These DRMs contain other proteins likely indirectly related to rubber biosynthesis (Figure 4B1,B2). The RTase and associated proteins (Figure 4C), are responsible, respectively, for substrate binding, catalysis, and compartmentalization and metabolic “balancing” (i.e., removal of metabolic byproducts of chain synthesis), and, perhaps, continued polymerization and eventual chain termination (RTase associated proteins).

Before polymerization can occur, two Mg^2+^ ions bind to an IPP anion to form the IPP-Mg complex, which is the true rubber polymer monomer [39,40,41,42]. Mg^2+^ ions are also required for FPP binding (or any other of the various allylic pyrophosphates capable of initiating rubber biosynthesis), but these Mg^2+^ ions do not have to bind to FPP for binding site recognition and binding, although they have to be in place before catalysis [39,40,41]. IPP-Mg and FPP or FPP-Mg then bind to the two small substrate-binding proteins (1.79 kDa and 3.99 kDa), although we do not yet know which substrate binds to which binding protein [37]. The CPT catalytic protein is held in an appropriate position by the dimeric protein scaffold, and the FPP/Mg/binding protein unit associates with the catalytic site of the CPT to deliver the allylic pyrophosphate initiator. A *Hevea* CPT binding protein ortholog did not appear in our guayule proteomic analysis. The IPP/Mg/binding protein unit can only then associate with CPT and deliver the IPP-Mg to the catalytic site. The CPT catalyzes chain elongation by a condensation reaction which forms the first *cis* polymerization of IPP to FPP, possibly through carbocationic polymerization [43], switching the nonallylic double bond of IPP to the allylic carbon, forming a new active end of the rubber chain. This catalytic reaction releases protons, pyrophosphate, and phosphate ions [24]. The initial *trans* C15 FPP is then shuttled partly through the channel of the RTase complex [3,19]. At this point, the IPP binding protein (or a different one) forms another IPP/Mg/binding protein unit, which then delivers a second IPP to the CPT for attachment to the active end of the elongating rubber chain. This reaction is repeated about 15,000 times in the formation of a high-molecular polymer (M_w_ of at least 1 million g/mol). After the first five condensation events, the original FPP molecule is entirely inside the rubber particle, and throughout polymerization, in *P. argentatum*, only the end five units interact with RTase itself (the active C5 end interacts with the catalytic site of CPT and the other four isopentenyl units [in *cis* configuration] with the hydrophobic RTase channel to the amorphous hydrophobic particle interior complex) [3,19]. It is also clear that each RTase has six active sites and these are arranged on the scaffold in a ring, forming the channel to the interior. Only one site is drawn in detail (Figure 4C), but the six are illustrated as the figure zooms out (Figure 4A,B1,B2) and the lipid rafts/DRMs come into view (Figure 4A,B1,B2).

**Figure 5:**
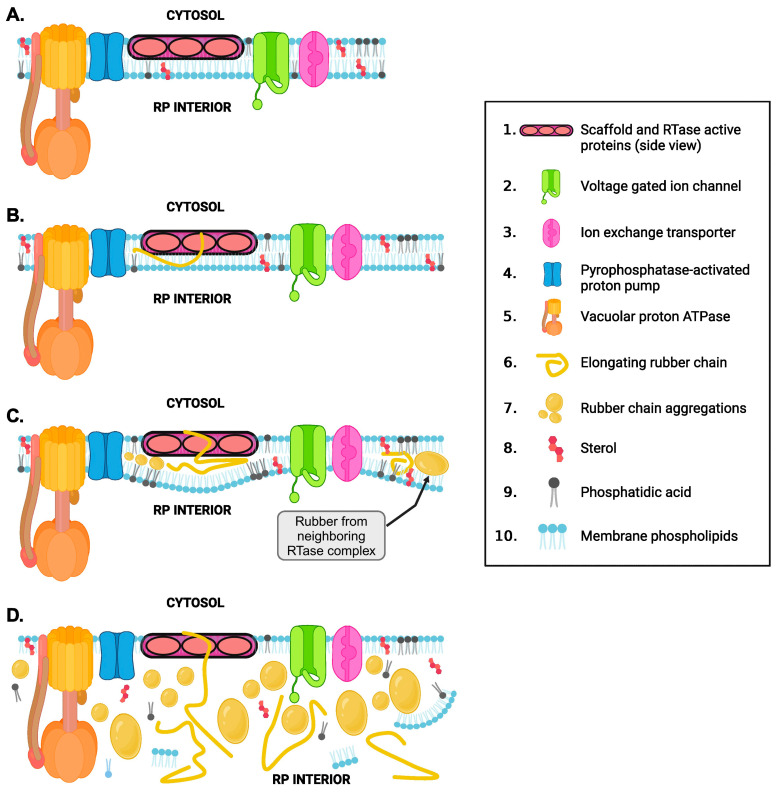
Evolution of rubber particle membrane around the putative RTase complex and stabilization of transmembrane protein components. (**A**). The nascent RP membrane is a bilayer into which the RTase complex members are integrated. (**B**). New rubber molecules are extruded into the hydrophobic interior of the lipid bilayer as they are synthesized. (**C**). Increased aggregations of rubber molecules accumulate between the fatty acid tails of the liquid bilayer, causing the bilayer to balloon out, resulting in separation of the lipid-bilayer leaflets. (**D**). As more hydrophobic rubber molecules accumulate, the bilayers separate entirely, resulting in the formation of a unilamellar vesicle. The hydrophobic environment of the RP interior serves to stabilize the hydrophobic residues on the alpha helices of the transmembrane proteins.

In our model (Figure 4), the large dimeric scaffold (monomer is 387 kDa) [37] holds the proteins critical to making the rubber polymer in place and forms the hydrophobic channel to the particle interior [24]. However, other closely associated proteins, such as the allene oxide synthase-like P450 (RPP proteins) and SRRP proteins [14,24,37] (not shown in Figure 4) may interact with the RTase, helping to stabilize the complex and support continued chain elongation [38,44]. However, this is not the only mechanism likely to stabilize complex members, as the rubber interior of the particle also likely stabilizes this membrane complex, as detailed in Figure 5. The nascent RP membrane is a bilayer into which RTase complex components are integrated (Figure 5A). While several of these components are large enough to span the membrane bilayer (ion transporters, ATPases, etc.), the core rubber synthetic machinery spans only the outer leaflet of the membrane. As rubber synthesis is initiated, rubber chains are extruded into the interior of the membrane into the space between outer and inner membrane leaflets (Figure 5B). As rubber synthesis continues, aggregations of rubber molecules between the lipid bilayers push the membrane leaflets apart (Figure 5C), causing the membrane to bulge and “balloon.” Eventually, accumulations of rubber chains push the membranes entirely apart, resulting in the formation of a unilamellar vesicle/rubber particle (Figure 5D). The hydrophobic interior of the RP generated by the accumulation of rubber molecules now serves to stabilize the hydrophobic moieties of the alpha helices of larger complex members (ATPase, ion transporters) which had previously been stabilized by the fatty acid chains of the original inner membrane leaflet. The hydrophobic RP interior also helps stabilize the interaction of RTase members with one another.

Among the other proteins we have putatively identified in association with the RTase (Figure 4C), the putative long-chain acetyl-CoA synthetase may activate membrane-derived fatty acids, which are then used to modify the rubber polymers so that they remain soluble in the rubber particle interior even at molecular weights where rubber alone would be a rigid solid. The rubber particle interior contains considerable low-molecular-weight material, the origin of which is unclear, but which maintains core fluidity [9,45]. Also, as the synthesis of rubber molecules proceeds at the catalytic site of the RTase complex, the hydrogen and pyrophosphate ions generated during catalysis gradually accumulate in the inner surface of the membrane adjacent to the allene oxide synthase-like/RPP proteins (Figure 4C). If they continued to build up, the RTase environment would acidify and inhibit or halt polymerization. We suggest that the RTase-associated pyrophosphate-energized proton pump hydrolyzes excess pyrophosphate transports protons away from the RTase and into the interior of the rubber particle, preventing localized cytosolic acidification. The pyrophosphatase-activated proton pump identified in the RP membrane exhibits significant similarity to the vacuolar proton pyrophosphatase AVP1, which functions in vacuolar acidification in *Arabidopsis* and which participates in the regulation proton/pH gradients involved in auxin transport [46,47]. RTase-associated ATPases further contribute to maintaining proton and ATP homeostasis across the rubber particle membrane, although the contribution of these complexes to RTase function requires further investigation. For example, the orientation of these complexes in the RP membrane remains to be defined. Since vacuolar ATPases pump protons into the exterior of the vacuole and plasma membrane ATPases pump protons into the apoplastic space, both of which are functionally contiguous with the lumen of the ER, where RPs originate, we have made the initial assumption that these transporters are oriented to pump protons into the interior of the RP. However, it is possible that these pumps may be oriented to pump protons out of the RP into the cytosol. In this case, ATP could be provided to the proton ATPases by the ATP/ADP antiporter detected in the RP membrane. Regardless of the orientation of the proton ATPase, the prevalence of proteins involved in proton transport indicates that localized proton gradients and pH is likely to be critical for RTase activity and long-chain rubber synthesis.

Ultimately, high-molecular-weight rubber may be released from the RTase partly by physical constraints [39,48], or it could be displaced by high concentrations of allylic pyrophosphate or protons [40,41,49]. In this case, as the rubber chain continues to elongate, sustained build-up of metabolic by-products eventually overwhelms the capacity of the pyrophosphatase-activated proton pumps, ATPases, and ion channels, resulting in termination of rubber chain elongation and dissociation of the rubber chain from the *cis*-prenyl transferase-allene oxidase/P450 protein-chaperone complex.

## 4. Materials and Methods

### 4.1. Isolation of Active Rubber Particles from Parthenium argentatum

Enzymatically active washed RPs (WRPs) were purified from the bark parenchyma of freshly harvested *P. argentatum*. WRPs were prepared twice from cold-induced plants harvested in the winter of 2013–2014, and again in the winter of 2014–2015. The plants were the generous gift of Dr. L. Johnson, PanAridus LLC, Casa Grande, AZ, USA (no longer operating). Freshly harvested plants were shipped overnight in coolers with wet ice. Buffers (extraction buffer and wash buffer) were prepared the day before the extraction and stored at 4 °C. Extraction buffer was 100 mM Tris pH 7.5 at room temperature (pH 7.8 at 4 °C), 5 mM MgSO_4_, 0.2% (*m*/*v*) BSA, 1% (*v*/*v*) ascorbic acid, pH 7.5. Immediately before use, 0.1 mM phenylmethanesulfonyl fluoride [PMSF], 0.1 mM pefabloc, 5 mM β-mercaptoethanol, 0.0025% [*v*/*v*] antifoam A, and 7% [*w*/*v*] pre-cooled polyvinylpolypyrrolidone [PVPP] were added to the initial extraction buffer, and the complete buffer was maintained at 4 °C. Initial wash buffer consisted of 100 mM Tris pH 7.5, 5 mM MgSO_4_, 0.2% (*m*/*v*) BSA, and was cooled overnight at 4 °C. Then, 0.1 mM PMSF, 0.1 mM pefabloc, and 5 mM DL-dithiothreitol were added to the wash buffer immediately prior to use, after which the buffer temperature was maintained at 4 °C. Membrane-grinding buffer was prepared as described previously [32].

Guayule branches were defoliated by flash freezing in N_2_(l), followed by a blow with an ice bucket to remove embrittled leaves [50]. The bark was peeled from each stem and immediately immersed in ice-cold aqueous 1% ascorbic acid (added as an antioxidant). Bark was drained using a flour sieve and ground for 2 min in extraction buffer (3:1 buffer to bark *w*/*w*) using a Waring blender (1 L or 1 gallon depending on specific sample size) [51]. Rubber particles were then isolated from the liquid phase as described [28], except that the second washing step was performed in membrane-grinding buffer [32] rather than wash buffer. The sample temperature was kept at 4 °C for all steps, and membrane (RP membrane and detergent-resistant membrane microdomain) sample mass and volume were measured and recorded at each step.

### 4.2. Purification of Washed Rubber Particle Membranes

WRP membranes were isolated using protocols modified from [27]. WRPs in membrane-grinding buffer [32] were centrifuged at 100,000× *g* at 4 °C for 50 min (SW32Ti rotor, Beckman Coulter Optima^TM^ L-90K Ultracentrifuge, Brea, CA, USA). The layer of RPs which floated to the top of the buffer was coagulated by gently pipetting glacial acetic acid onto the surface in a dropwise fashion and then immediately removing the coagulated latex with a small spatula and tweezers. The supernatant from each tube was then decanted and stored at −80 °C. RP membranes (precipitated at the bottom of the tubes) were resuspended in membrane resuspension buffer, which contained 10 mM BTP-MES [bis-tris propane and 2-(N-morpholino)ethanesulfonic acid], 250 mM sucrose, and 20% (*m*/*v*) glycerol, to which the following protease inhibitors were added immediately prior to use: 200 ng/mL leupeptin, 100 µM PMSF, 200 µM benzamide/benzamidine, 200 µM pepstatin A, and 10 µM poritinin. The resuspended WRP membranes were spun at 100,000× *g* at 4 °C for 50 min (SW55Ti rotor; Beckman Coulter, Brea, CA, USA). This step was repeated twice, after which the isolated RP membranes were resuspended in membrane resuspension buffer, flash-frozen in liquid nitrogen, and stored at −80 °C.

### 4.3. Purification of Detergent-Resistant Membrane Microdomains

Detergent-resistant membrane microdomains were isolated using protocols detailed in [52]. Briefly, WRP membranes were prepared initially as described above (Section 2.2). However, during the RP membrane resuspension steps, membranes were resuspended in a modified (glycerol-free) resuspension buffer. After the first resuspension step, samples were split in half, and one-half of the membranes were spun down and washed and with normal (resuspension buffer with 20% glycerol), resuspended in the same buffer containing 20% glycerol, then flash-frozen in liquid nitrogen. The second half of the sample was spun down and resuspended in TNE buffer (25 mM Tris pH, 150 mM NaCl, 5 mM EDTA, pH 7.5 with 0.1% Triton-X 100) (reagents from Sigma-Aldrich, St. Louis, MO, USA). These samples were then layered on top of ultracentrifuge tubes containing a slab sucrose gradient of (from bottom to top) 2.4 M, 1.6 M, 1.4 M, and 0.15 M sucrose prepared in TNE-100. Samples were centrifuged at 100,000× *g* at 4 °C for 18 h (SW55Ti rotor). Each layer of the gradient was then separately harvested using a pipette and placed in a fresh ultracentrifuge tube to which five volumes of TNE-100 (TNE buffer containing 0.1% Triton-X100) were added. Samples were recentrifuged at 100,000× *g* at 4 °C for 50 min (SW55Ti rotor). Precipitated RP detergent-resistant membrane microdomains (DRMs) were resuspended in TNE-100 buffer (without DTT). DRMs were flash-frozen in liquid nitrogen and stored at −80 °C. The sample temperature was kept at 4 °C for all steps, and the membrane sample mass and volume were measured and recorded at each step.

### 4.4. Phospholipid and Sterol Extractions

To determine the phospholipid composition of RP membranes and DRMs, phospholipids were extracted from four biological replicates of RP membrane and DRM fractions. Samples were extracted using 400 µL CHCl_3_:MeOH:12 M HCl (50: 100: 1, *v*/*v*) per 50 µL resuspended membrane fraction. Extracts were partitioned into two phases by adding 400 µL CHCl_3_ and 200 µL of 0.9% (*m*/*v*) NaCl. The organic phase was collected from each tube and washed by partitioning against CHCl_3_:MeOH:1 M HCl (3:48:47, *v*/*v*/*v*). Organic phases were collected and dried under nitrogen gas. Extracts were re-dissolved in 100 µL CHCl_3_, and samples were separated using thin-layer chromatography (TLC) and visualized with 5% primuline solution (dissolved in 80% acetone), as described below (Section 4.5). A mixture of authentic phospholipids commonly found in plant membranes (Avanti Polar Lipids, Inc., Alabaster, AL, USA) provided reference standards. Sterols were extracted from three biological replicates of RP membrane and DRM fractions. Samples were extracted using 400 µL CHCl_3_:MeOH (70:30 *v*/*v*) per 50 µL membrane fraction. The CHCl_3_:MeOH extraction solvent contained deuterated internal standards cholesterol-d7, β-sitosterol-d6, and campesterol-d6 (Avanti Polar Lipids, Inc., Alabaster, AL, USA) at concentrations of 1 µg/µL for each sterol. Samples were sonicated at 60 Hz at room temperature for 30 min, and then centrifuged at 8000× *g* for 10 min The organic phase was collected and dried under nitrogen gas. Samples were resuspended in 100 µL of CHCl_3_:MeOH (70:30, *v*/*v*), filtered, and analyzed by LC-MS/MS.

### 4.5. Thin-Layer Chromatography

Thin-layer chromatography of rubber particle membrane phospholipids was completed at the Laboratory for the Analysis of Metabolites for Plants (LAMP) metabolomic facility at The Ohio State University. TLC plates (silica gel 60 plates, 20 cm × 20 cm × 0.2 cm, EMD-Millipore; MilliporeSigma, Burlington, MA, USA) were activated 48 h in advance by dipping them for 10 s in the impregnating solution: 1% (*w*/*v*) potassium oxalate and 2 mM EDTA in MeOH:H_2_O (2:3 *v*/*v*). The soaked plates were then air-dried and stored at 115 °C until use. Phospholipid extracts were spotted on the activated plates. Dots were placed 2 cm from the bottom and side edges and 0.7 cm apart from each other. TLC tanks (as well as the filter paper placed on the inside of three sides of the tank to maintain chamber saturation) were washed with methanol, chloroform, and separation solvent. Phospholipids were separated using an alkaline separation solvent: CHCl_3_:MeOH:25% NH_4_OH:H_2_O (90:70:4:16, *v*/*v*/*v*). After separation, plates were air-dried in a fume hood (2–5 min maximum drying time) and sprayed with 5% primuline solution (dissolved in 80% acetone).

### 4.6. Phospholipid and Sterol Identification and Quantification Using LC-MS/MS

Phospholipid and sterol quantifications were performed at the LAMP metabolomic facility at The Ohio State University. Prepared phospholipid extracts were injected into an Agilent 1260 HPLC coupled to a 6460 Triple Quadrupole LC/MS/MS (Agilent Technologies, Santa Clara, CA, USA). Protocols for separating and identifying phospholipids were modified from previously published protocols [53]. Briefly, target phospholipids were separated using an Agilent Poroshell EC-C18 column (50 mm × 3 mm, 2.7 µm, Agilent Technology, Santa Clara, CA, USA) at 50 °C. H_2_O with 0.2% formic acid was used as solvent A, and methanol with 0.2% formic acid was used as solvent B. The solvent gradient was as follows: 80–100% (0–10 min); 100% (11–15 min); 100–80% (16 min); 80% (17–20 min), with a flow rate of 0.4 mL/min Compounds were ionized using electrospray ionization (ESI), and the spray chamber settings were: gas temperature at 300 °C, gas flow at 10 L/min, nebulizer pressure at 35 psi, sheath gas temperature at 350 °C, sheath gas flow at 12 L/min, and capillary voltage at −2250 V.

Sterol samples were analyzed using the same LC-MS/MS system and column as for phospholipid analyses. The column was incubated at 30 °C and compounds were eluted from the column using an isocratic flow of 0.1% acetic acid in methanol at 0.45 mL/min. Compounds were ionized using Atmospheric Pressure Chemical Ionization (APCI). The spray chamber settings were: gas temperature at 325 °C, vaporizer temperature at 350 °C, gas flow at 10 L/min, nebulizer pressure at 35 psi, and capillary voltage at +4000 V. Chromatographic data for both sterols and phospholipids were analyzed using Agilent Mass-Hunter software (version B.06.00 SP01, build 6.0.388.1).

### 4.7. Proteomic Studies

#### 4.7.1. Detergent Solubilization of Proteins from Rubber Particle Membranes and Rubber Particle DRM Fractions

A series of detergents were tested for their ability to release proteins from the RP membrane and DRM fractions, including Nonidet P-40 substitute, Brij-35, β-methylcyclodextrin, and deoxycholate. Proteins were quantified from three independently prepared RP membranes and DRM fractions using the bicinchoninic protein assay (BCA; Pierce-Endogen, Thermo Scientific, Rockford, IL, USA). Proteins extracted from detergent-solubilized and non-detergent-solubilized RP membrane and DRM samples were separated via SDS-PAGE as described previously [32]. Protein sequencing was performed via LC-MS/MS at The Ohio State University Campus Chemical Instrumentation Center (CCIC).

#### 4.7.2. 1-D Gel Separation of Peptides and SDS-PAGE Gel Processing

Firstly, 20 µg of each sample was diluted with membrane resuspension buffer to a final volume of 25 µL, after which 6.25 µL of 5X Laemmli buffer (315 mM Tris pH 6.8, 60% [*v*/*v*] glycerol, 5% [*m*/*v*] SDS, 100 mM DTT; reagents from Sigma-Aldrich, St. Louis, MO, USA) was added to each sample. Samples were then loaded onto 4–15% gradient polyacrylamide gels and separated via SDS-PAGE at 150 V for 65 min. Gels were then Coomassie stained and individual lanes were excised. Each lane (one lane per sample) was separated into 8 fractions of equal length, from top to bottom. Individual gel fragments were diced into small cubes and transferred into 96-well plates (one well per fragment). Digestion and extraction were performed using the Ettan Spot Handling Workstation 2.1. Gel pieces were washed in 100 µL of a 50:50 methanol:5% acetic acid solution for 30 min, followed by a 10 min acetonitrile wash. Samples were then dried-down and resuspended in 50 mM ammonium bicarbonate solution containing 5 mg/mL DTT for 30 min at room temperature. Ammonium bicarbonate–DTT solutions were aspirated away, and gel fragments were then suspended in 50 mM ammonium bicarbonate 15 mg/mL iodacetamide and incubated for 30 min at room temperature in the dark. This solution was then aspirated away from the fragments and the gel pieces were incubated in 50 mM ammonium bicarbonate for 10 min, followed by 10 min of incubation in acetonitrile, and this process was repeated twice. Following the final incubation, gel fragments were taken to complete dryness.

#### 4.7.3. Trypsin Digestion of Peptides in Gel Fragments

Dried gel slices for each sample were rehydrated in 25 µL of 50 mM ammonium bicarbonate. Following rehydration, 75 µL of sequencing-grade modified trypsin (Promega, Madison, WI, USA) prepared at a concentration of 10 µg/mL in 50 mM ammonium bicarbonate was added to each sample, and the trypsin mixture was driven into the fragments at 37 °C for 15 min. Then, 25 µL of 50 mM ammonium bicarbonate was added to each sample, and digestion of peptides was allowed to occur for 6 h at 37 °C. Following digestion, peptides were extracted from the polyacrylamide matrix by eluting samples three times in 50 µL 50:50 acetonitrile:acetic acid, after which a final extraction of 50 µL acetonitrile was performed. Peptide extracts were pooled together and dried in a vacuum centrifuge, and then each sample was resuspended in 28 µL of 50 mM acetic acid in water.

#### 4.7.4. LC-MS/MS Conditions for Proteomic Analyses

Capillary liquid chromatography tandem mass spectrometry (capillary-LC/MS/MS) for global protein identification was performed on a Thermo-Fisher LTQ Orbitrap mass spectrometer equipped with a microspray source (Michrome Bioresources, Inc., Auburn, CA, USA) operated in positive ion mode. Samples were separated on a capillary column (0.2 × 150 mm Magic C18AQ, 3 µ 200 A, Bruker Daltronics, Billerica, MA, USA) using an Ultimate^TM^ 3000 HPLC system (Thermo Scientific, Waltham, MA, USA). Each sample was injected into the µ-Precolumn Cartridge (Thermo Scientific) and desalted with 50 mM acetic acid for 5 min. The injector port was then switched to inject and the peptides were eluted off of the trap onto the column. Mobile phase A was 50 mM acetic acid in water, acetonitrile was used as mobile phase B, and the flow rate was set at 2 µL/min. Mobile phase B was increased from 2% to 50% in 30 min and from 50–90% in 5 min, and then kept at 90% for another 2 min before being brought back to 2% in 1 min. The column was equilibrated at 2% of mobile phase B (or 98% A) for 20 min prior to the next sample injection. MS/MS data were acquired with a spray voltage of 2.2 KV and a capillary temperature of 175 °C. The scan sequence of the mass spectrometer was based on the preview-mode data-dependent TopTen™ method: the analysis was programmed for a full scan recorded between *m*/*z* 350 and 2000 and an MS/MS scan to generate product ion spectra to determine the amino acid sequence in consecutive scans of the five most abundant peaks in the spectrum. To achieve high-mass-accuracy MS determination, the full scan was performed in FT mode and the resolution was set at 60,000. The AGC target ion number for the FT full scan was set at 1 × 10^6^ ions, the maximum ion injection time was set at 1000 ms, and the micro scan number was set at 1. MSn was performed using ion trap mode to ensure the highest signal intensity of MSn spectra. The AGC target ion number for the ion trap MSn scan was set at 10,000 ions, the maximum ion injection time was set at 50 ms, and the micro scan number was set at 1. The CID fragmentation energy was set to 35%. Dynamic exclusion was enabled with a repeat count of 1 within 18–36 s (depends on the length of the gradient), a mass list size limit of 500, an exclusion duration of 12–20 s (depending on the length of the gradient), and a low mass width and high mass width of 30 ppm. When the sample was digested with trypsin, an exclusion list containing major trypsin autolysis peptides was applied so these peaks would not be detected. The reject mass width window was set at 30 ppm.

#### 4.7.5. Proteomic Data Analysis

Sequence information from the MS/MS data was processed by converting the “.raw” files into a merged file (.mgf) using an in-house program, RAW2MZXML_n_MGF_batch (merge.pl, a Perl script). Isotope distributions for the precursor ions of the MS/MS spectra were deconvoluted to obtain the charge states and monoisotopic m/z values of the precursor ions during the data conversion. The resulting “.mgf” files were searched using Mascot Daemon by Matrix Science version 2.3.2 (Boston, MA, USA) and the database was searched against the most recent NCBI databases. The mass accuracy of the precursor ions was set to 20 ppm, and an accidental pick of 1 13C peaks was also included into the search. The fragment mass tolerance was set to 0.5 Da. The considered variable modifications were oxidation (Met), deamidation (N and Q), and carbamidomethylation (Cys). Four missed cleavages for the enzyme were permitted. A decoy database was also searched to determine the false discovery rate (FDR), and peptides were filtered according to the FDR. The significance threshold was set at *p* < 0.05, and bold red peptides were required for valid peptide identification. Proteins with a Mascot score of 50 or higher with a minimum of two unique peptides from one protein having a -b or -y ion sequence tag of five residues or better were accepted. Any modifications or low-score peptide/protein identifications were manually checked for validation. To identify putative proteins, peptide sequences were analyzed using Mascot software (versions 4.8.4-4.9.0) and BLASTed against the NCBI (National Center for Biotechnology Information) genomic/proteomic databases and the guayule transcriptome generated by us in parallel to this investigation.

## 5. Conclusions

- The rubber particle membrane has a distinct phospholipid composition primarily made up of detergent-resistant membrane microdomains (up to 90% of the total membrane fraction).
- Proteomic analyses indicate that the detergent-resistant membrane microdomains present in rubber particle membranes contain proteins putatively involved in rubber synthesis.
- The rubber particle membrane is enriched in proteins putatively involved in metabolically balancing rubber chain synthesis by extruding the by-products of rubber synthesis (protons, pyrophosphate, and ions) from the interior of the rubber particle into the cytoplasm.
- We propose a new model of the RTase complex.

## Figures and Tables

**Table 1:** Amounts of microsomal membranes and DRMs present in washed rubber particles: RPs were isolated from guayule parenchyma cells, and aliquots of RPs were collected and weighed (rubber particle mass, g). As noted in Figure 1, following extraction from RPs, the total membrane fraction was weighed (RP membrane mass, mg), then resuspended and split into two sub-fractions. One of these sub-fractions was weighed (RP mass for DRM isolation, mg), treated with Triton X-100, and DRMs were isolated from the resulting suspension. DRMs were then precipitated and weighed (DRM mass, mg). To determine the contribution of membranes to the RP mass, the total percentage of the RP mass consisting of membrane components (Membrane % of total RP mass) was then calculated using the following formula: ([RP membrane mass, mg]/[RP particle mass, g × 1000]) × 100. To determine the contribution of DRMs to the membrane structure of the RP, the percentage of the total RP membrane mass made up of membrane components (DDRM % of initial RP mass) was then calculated using the following formula: ([DRM membrane mass, mg]/[RP membrane mass for DRM isolation, mg]) × 100.

	RP Total Membrane Yield			Detergent-Resistant Membrane Yield		
Sample	Rubber Particle Mass (g)	RP Membrane Mass (mg)	Membrane % of Total RP Mass	RP Membrane Mass for DRM Isolation (mg)	DRM Mass (mg)	DRM % of RP Membrane Mass (mg)
1	15.45	203.7	1.310	135.8	115.2	84.83
2	8.228	104.4	1.269	69.6	39.6	56.90
3	7.782	197.1	12.533	131.4	71.5	54.41
1	2.195	58.1	2.647	37.2	20.8	55.91
2	4.627	74.2	1.604	47.4	18.7	39.45
3	2.598	67.3	2.590	39.8	27.1	68.09
4	3.965	57.0	4.315	31.9	20.3	63.64
5	6.512	41.4	1.907	23.4	21.2	90.60

**Table 2:** Quantification of proteins from RP membrane and DRM fractions using the bicinchoninic protein assay (BCA).

Microsome Fraction		DRM Fraction	
Sample ID	µg/µL	Sample ID	µg/µL
M-1	78.50	D-1	35.82
M-2	36.10	D-2	15.22
M-3	41.61	D-3	19.05

## Data Availability

Data are available upon request from the corresponding authors due to intellectual property restrictions arising from the industrial–academic collaboration responsible for the research.

## Supplementary Materials

The following supporting information can be downloaded at: https://www.mdpi.com/article/10.3390/plants13212970/s1, Table S1: Phospholipid standards used in TLC and LC-MS/MS analyses; Table S2: BLAST analyses of guayule rubber particle membrane proteomic data.
